# What Does Quality of Care Mean in Lower-Grade Glioma Patients: A Precision Molecular-Based Management of the Tumor or an Individualized Medicine Centered on Patient’s Choices?

**DOI:** 10.3389/fonc.2021.719014

**Published:** 2021-07-20

**Authors:** Luc Taillandier, Tiphaine Obara, Hugues Duffau

**Affiliations:** ^1^ Centre de Recherche en Automatique Nancy France - UMR 7039 - BioSiS Department, Faculty of Medicine, Université de Lorraine, Vandoeuvre-lès-Nancy, France; ^2^ Neurology Departement, Neurooncology Unit, CHRU, Nancy, France; ^3^ Department of Neurosurgery, Gui de Chauliac Hospital, Montpellier University Medical Center, Montpellier, France; ^4^ Team “Plasticity of Central Nervous System, Stem Cells and Glial Tumors”, National Institute for Health and Medical Research (INSERM), U1191 Laboratory, Institute of Functional Genomics, University of Montpellier, Montpellier, France

**Keywords:** glioma, quality of life, evidence-based medicine, precision medicine, awake surgery, chemotherapy, radiation therapy

## Introduction

Neurooncology is a young specialty which initially dealt mostly with glioblastoma patients with a short overall survival (OS). Yet, recently the scope gradually expanded by taking care of lower-grade glioma (LGG) patients with a longer OS (1). Historically, these patients were managed with a “wait and see attitude” claiming “benignity” despite 6 to 7 years OS (2, 3). Therefore, quality of care was mainly based on physician’s subjectivity and not on the natural history, leading to beliefs that early surgery was not adapted due to “normal neurological examination”.

Twenty years later, it is now admitted that (i) beyond seizures, LGG patients suffer from cognitive and behavioral deficits at diagnosis even in incidental cases (4) (ii) this tumor will inescapably transform in higher grades, explaining the use of “lower-grade glioma” (mixing II/III) expression (5) (iii) early surgery is a main therapeutic factor (significant correlation between extent of resection and OS) (6, 7) (iv) early radiotherapy, at least given alone, is not associated with decreased mortality (6, 8). These changes resulted in a longer life expectancy now over 15 to 16 years (9–11).

Moreover, neurooncologists had to pay more attention to quality of life (QoL) for patients who must learn to live with a chronic neoplastic disease.

On the other hand, because LGG will systematically recur, further adapted treatments have to be administrated (12). However, heterogeneity of progression patterns (13) makes the prediction of timescales of proliferation, migration, and degeneration at the individual level impossible.

To provide more reliable prognostic factors, advances in molecular biology led to a new classification designed for more appropriate decisions (14). Surprisingly, although genetics was initially a tool to better dissociate types of LGG with distinct prognosis, molecular biology rapidly became the first parameter in guidelines (15). Although useful, by taking mostly account of genetics criteria and extrapolating a correlation to specific OS based upon statistical analysis, there is a risk to neglect tumor-host interactions, patient’s wishes, and long-term QoL.

Here, the main purpose is to redefine what “best quality of care” means by considering both tumor characteristics and patient’s personal criteria. The ultimate goal is to give the choice of therapeutic orientation at each step thanks to honest although complex and time-consuming information highlighting oncofunctional balance and various strategies individually adapted over time in parallel with changes in tumor behavior and patient’s expectations.

## Toward Hegemony of Precision Medicine Based on Glioma Molecular Profile: The Risk to Impose a “Unique Solution”

Official guidelines, elaborated on EBM and mostly relying on randomized controlled trials (RCTs), were primarily designed to help physicians within a framework facilitating decision making and thus defining a “quality of care”.

Particularly, progress allowed a refinement of the WHO classification increasingly based on genetic profiling (14, 16). This praiseworthy initiative gradually drifts toward more drastic molecular recommendations. Such a so-called precision EBM (17), glioma, and not patient-centered, is questionable. First, the 2016 classification (14) was built on few parameters (e.g., 1p19q, IDH, and MGMT status) too simplistic to capture complex glioma behavior and host interactions. Because improved knowledge will still take a considerable time, it is difficult to understand how “quality of care” can be determined on preliminary criteria. For example, IDH wild-type glioma were considered as molecular glioblastoma (15), whereas by integrating markers, such as TERT or EGFR, distinct groups exhibiting different prognosis (18–20) are now identified. Thus, many patients dogmatically receive and continue to receive RT-CT, whereas it would be more adapted to follow some of them by integrating parameters, such as growth rate (21, 22), and wonder about the multimodal heterogeneity. Similarly, because response rate to CT is statistically higher in oligodendrogliomas, it was peremptorily postulated that upfront, CT was not indicated in astrocytomas by neglecting that stabilization or shrinkage was nonetheless possible (23), thus opening the door to surgery which can have a major impact on prognosis. Thereby, tumor genetics represent an important but not exclusive part of the story (24).

These examples illustrate the drift in the utilization of EBM originally defined as “the conscientious, explicit, and judicious use of the current best evidence in making decisions about the care of individual patients” (25). Yet, the power of population-based observational studies based on real-life data collected in clinical routine was progressively denied for the benefit of exclusive RCTs. Nevertheless, they suffer from serious limitations (26, 27), first the inclusion of selected patients not reflecting the daily practice [e.g., young age in Stupp et al. trial (28)], or the fact that factors like extent of resection are overlooked (29), whereas a meta-analysis confirmed a strong correlation to OS (7) even after adjustment for molecular markers (6). Currently, a statistical result identified by RCTs is erected as a rigid law to be applied to each patient, without considering the inter-subject multimodal variability (30). If RCTs are the most convincing and effective strategy for answering a simple therapeutic question with measured short-term effects, they remain unsuitable to the current neuro-oncological issues. Indeed, the challenge in this era is rather to know what kind of patients will respond effectively to a therapeutic strategy and not to determine the best treatment among highly selected patients. Even if statistical tools as interaction tests used in RCTs design could give results of subgroup analyses, they remain insufficient because of a lack of statistical power and never allow conclusion. In fact, when the clinical questions and situations are not compatible with the use of RCTs, the importance of observational studies should be reconsidered. If they are conducted with a methodological rigor (long follow-up, sufficient size, few missing data) and analyzed with statistical tools limiting biases, they could provide reliable evidence and enable a better understanding of the long-term evolution. Besides, a Cochrane review (31) highlighted that the results of observational studies and RCTs are most often in agreement.

Third, EBM was not designed to validate a multistep strategy over years. Indeed, time-scales are different between the long life expectancy of patients and many RCTs with only a short follow-up which optionally use surrogates (such as progression-free survival [PFS] moreover often not accurately assessed) to demonstrate within the time allowed a significant difference regarding investigated parameters. This “reality of the moment” does not reflect long-term OS and QoL, e.g., early RT may have an impact on PFS but not on OS (8) while generating delayed and sometimes major cognitive deterioration (32, 33) not observed with too short a follow-up. It was the case in the RCT trial by Buckner et al. (29) within which (i) contrast enhancement was noted for approximately 50% of patients which is quite atypical for LGG (ii) surgical status is mainly represented by biopsies or partial surgeries in opposition to specialized teams practices mainly carrying out subtotal or total resections (iii) IDH status is only accessible in less than half of the cases and 1p19q in a quarter of them (iv) and cognitive analysis was only based on the MMSE (designed for dementia patients) with a longitudinal partial completion (**Table 1** for a critical review of RCTs).

**Table 1:** Main RCTs in medical neurooncology for LGGs: critical review of cognition and quality of life data.

Reference	Authors conclusion	Cognition	Quality of Life
Klein M et al. Neuro-Oncology 2021;23:803–11 (34)	“Neuropsychological assessment was performed in 98 patients (53 RT, 46TMZ). At 12 months, compliance had dropped to 66%, restricting analyses to baseline, 6 months, and 12 months. At baseline, patients in either treatment arm did not differ in memory functioning, sex, age, or educational level. Over time, patients in both arms showed improvement in Immediate Recall (*P* = 0.017) and total number of words recalled (Total Recall; *P* < 0.001, albeit with delayed improvement in RT patients (group by time; *P* = 0.011). Memory functioning was not associated with RT gross, clinical, or planned target volumes.	Memory functioning was assessed using the Visual Verbal Learning Test (VVLT). 12 months compliance 66% **No data beyond one year**	See Reijneveld JC et al. Lancet Oncol. 2016;17:1533-42. (35)
Breen WG et al. Neuro Oncol.2020;22:830-37 (36)	*“Long-term follow-up indicates no benefit to high-dose over low-dose radiation for low-grade gliomas”.*	“Cognitive function appeared to be stable after radiation as measured by MMSE” 187/203 MMSE at base line Completion <50% at all time points **Only MMSE**	**No data**
Dirven L, et al. Int J Radiat Oncol Biol Phys. 2019;104:90-100. (37)	*“The brain target volume receiving focal radiation therapy in fractions of 1.8 Gy to a total of 50.4 Gy did not appear to be independently associated with HRQoL in high-risk patients with low-grade glioma in the short term, as opposed to tumor progression”.*	No data expect QLQ C30 BN 20 pre-selected “cognitive functioning”	QLQ-C30 and QLQ-BN20 4 preselected HRQoL scales (global health status, cognitive and social functioning, and fatigue)
Baumert BG, et al. Lancet Oncol. 2016;17:1521-32. (38)	*“Overall, there was no significant difference in progression-free survival in patients with low-grade glioma when treated with either radiotherapy alone or temozolomide chemotherapy alone. treatment choices”.*	**Only MMSE** See Reijneveld JC **et al.** Lancet Oncol. 2016 (35)	EORTC QLQC30 + BN 20 See Reijneveld JC et al. Lancet Oncol. 2016 (35)
Reijneveld JC et al. Lancet Oncol. 2016;17:1533-42. (35)	*“The effect of temozolomide chemotherapy or radiotherapy on HRQOL or global cognitive functioning did not differ in patients with low-grade glioma”.*	**Only MMSE** Completion 1 year •TMZ 74% •RT 67% 3 Years •TMZ 58% •RT 57% **No data after 3 years**	EORTC QLQC30 + BN 20 Completion 1 year •TMZ 68% •RT 59% 3 years •TMZ 50% •RT 54% **No data after 3 years**
Buckner JC et al N Engl J Med. 2016;374:1344-55 (29)	*“In a cohort of patients with grade 2 glioma who were younger than 40 years of age and had undergone subtotal tumor resection or who were 40 years of age or older, progression-free survival and overall survival were longer among those who received combination chemotherapy in addition to radiation therapy than among those who received radiation therapy alone”.*	**Only MMSE** See Prabhu RS **et al.**, 2014 (39)	**No data**
Prabhu RS et al. J Clin Oncol. 2014; 32:535–41 (39)	*“The MMSE is a relatively insensitive tool, and subtle changes in CF may have been missed. over RT alone for patients with low-grade glioma.* *…* *The addition of PCV chemotherapy to RT improves PFS without excessive CF detriment over RT alone for patients with low-grade glioma”.*	**Only MMSE** Completion •55% 2 years •57% 3 years •44% 5 years **No data after 5 years**	–
Shaw EG et al. J Clin Oncol.2012;30:3065-70. (40)	*“PFS but not OS was improved for adult patients with LGG receiving RT + PCV versus RT alone. On post hoc analysis, for 2-year survivors, the addition of PCV to RT conferred a survival advantage, suggesting a delayed benefit for chemotherapy”*	**No data**	**No data**
van den Bent MJ, et al. EORTC Radiotherapy and Brain Tumor Groups and the UK Medical Research Council. 2005;366:985-90. (8)	*“Early radiotherapy after surgery lengthens the period without progression but does not affect overall survival.* *Because quality of life was not studied, it is not known whether time to progression reflects clinical deterioration.* *Radiotherapy could be deferred for patients with low-grade glioma who are in a good condition, provided they are carefully monitored”.*	***“Quality of life was not studied”*** “*To investigate whether patients free from tumour progression had any neurological signs and symptoms, the neurological signs and symptoms at 1 year were analyzed in patients who were still progression-free at 2 years. The use of this subset ensures that the acute effects of treatment have subsided, and that patients who are already progressing at 1 year but have not yet been diagnosed with progression are excluded from the analysis. Post-hoc analysis found no differences between the two groups for cognitive deficit, focal deficit, performance status, and headache (data not shown)”*	
Brown PD, et al. Int J Radiat Oncol Biol Phys. 2004;59(1):117-25 (41)	*“The presence of an abnormal baseline MMSE score was a strong predictor of poorer progression-free and overall survival for patients with a low-grade glioma. The baseline MMSE should be considered in future prognostic scoring systems”*	**Only MMSE**	**No data**
Shaw E et al. J Clin Oncol. 2002;20:2267-76. (42)	*« This phase III prospective randomized trial of low- versus high-dose radiation therapy for adults with supratentorial low-grade astrocytoma, oligodendroglioma, and oligoastrocytoma found somewhat lower survival and slightly higher incidence of radiation necrosis in the high-dose RT arm. The most important prognostic factors for survival are histologic subtype, tumor size, and age. The study design of the ongoing intergroup trial in this population will be discussed.*	“Grade 3 to 5 radiation neurotoxicity (necrosis) was observed in seven patients, with one fatality in each treatment arm” See Brown PD et al., 2004 (41)	
Karim AB et al. Int J Radiat Oncol Biol Phys. 2002;52:316-24. (2)	*“Early postoperative conventional RT such as that used for this protocol appears to improve the time to progression or progression-free survival, but not overall survival, for patients with low-grade glioma”.*	See van den Bent MJ 2005 (8)	See van den Bent MJ 2005 (8)
Kiebert GM et al.Eur J Cancer.1998;34:1902-9 (43)	*« A quality of life (QoL) questionnaire consisting of 47 items assessing a range of physical, psychological, social, and symptom domains was included in the trial to measure the impact of treatment over time. Patients who received high-dose radiotherapy tended to report lower levels of functioning and more symptom burden following completion of radiotherapy. These group differences were statistically significant for fatigue/malaise and insomnia immediately after radiotherapy and in leisure time and emotional functioning at 7-15 months after randomization. These findings suggest that for conventional radiotherapy for low-grade cerebral glioma, a schedule of 45 Gy in 5 weeks not only saves valuable resources, but also spares patients a prolonged treatment at no loss of clinical efficacy”*		Since at the start of the study no well-validated, standardised QoL questionnaire was available for this population of patients, a questionnaire was constructed to meet the requirements of this study protocol. The questionnaire designed for this study was primarily adapted from a variety of sources including the Sickness Impact Pro^®^le (SIP), the Rand Corporation Health Insurance Study battery of questionnaires, the Center for Epidemiological Studies Depression Scale, and from previous questionnaires employed within the EORTC. A preliminary version of the questionnaire was pretested on a sample of patients at the Free University Hospital in Amsterdam, The Netherlands. The questionnaire consisted of 47 items assessing a range of physical, psychological, social, and symptom domains. Initial completion 82/345 pts. Completion à 36-60 months 61/143
Karim AB et al. Int J Radiat Oncol Biol Phys. 1996;36:549-56 (44)	*“The EORTC trial 22844 has not revealed the presence of radiotherapeutic dose-response for patients with LGG for the two dose levels investigated with this conventional setup, but objective prognostic parameters are recognized. The tumor size or T parameter as used in this study appears to be a very important factor”.*	“The sequelae and the quality of life do not appear to be different in the two arms but will be reported separately later in another report”	

Fourthly, whereas the quality of care relying on RCT depends on a reductionist panel of criteria, the selection of parameters “officially recognized” as decreed under the guise of EBM is questionable. For example, velocity expansion diameter is not incorporated in trials while it is an independent prognostic marker not correlated to molecular profile (45) and more reliable than 2007 WHO classification to predict OS (46, 47). Moreover, a main weakness of the 2016 WHO classification is arbitrarily to not consider intra-tumoral heterogeneity (19, 48, 49). Indeed, although areas of malignant transformation are frequently identified in the middle of LGG, especially after extensive surgery, they are not recognized as “foci of grade III/IV” within a grade II glioma but condition the final grading for the entire tumor. This oversimplification leads to a monolithic strategy, namely to administrate RT-CT, while efficient alternative exists, particularly to delay adjuvant treatments following maximal resection with a 95% survival rate at 5 years (50).

To sum up, due to a new orientation of EBM different from the Sackett et al. seminal concept (25) this “precision-medicine” risks to indirectly impose a “unique solution” based upon few molecular markers unable to reflect the complex glioma-host interactions. This simplistic inflexible attitude does not really represent the “informed consent” of the patient.

## The Alterative Way of Multimodal and Adaptive Individual Decision Making Aiming To Anticipate the Story Years in Advance

Because LGG patients live one to two decades, neurooncologists should learn to anticipate functional considerations. Indeed, a major lack of “precision-medicine” in gliomas is to prioritize analysis of PFS and OS as first endpoints at the expense of QoL. However, if a patient is doing well, this means that he/she is still alive, while the reverse is not true. Therefore, QoL should be more systematically considered as the main endpoint since LGG patients should have an active life (30). Yet, physicians are usually content with a basic neurological examination optionally with a simplistic neuropsychological assessment (e.g. MMSE) and a performance scale score (15). Nonetheless, to enjoy an optimal lifestyle (social investment, sexuality, childbirth, work) preservation of higher-order cognitive, emotional, and behavioral functions is mandatory (12). Neurosurgeons developed intraoperative awake mapping and monitoring of conation, cognition, and personality, resulting in a connectome-based resection according to a real-time investigation of neural networks and taking account of neuroplasticity (51–53). This led to a decrease of morbidity with stabilization or even improvement of postoperative neuropsychological scores (4) and over 97% of return to employment (54). By contrast, these types of high-level parameters have never been reported in CT/RT randomized study.

Beyond the lack of cognitive or QoL parameters framing each treatment in RCTs for LGG, and criticisms concerning tools (MMSE or QoL questionnaires tailored for malignant rapidly evolving tumors), these criteria are nonetheless essential to elaborate new guidelines paving the way for “quality of care.” Neuro-oncologists should ask the patient to define his/her own expectations and adjust the management accordingly (12, 33), e.g., awake surgery with identification of eloquent networks *à la carte* (55). Indeed, the patient must understand during the first meeting that therapeutic reserve is not inexhaustible. Typically, early RT may improve glioma control for years but entire re-irradiation is not possible at progression. This issue should be clearly explained to anticipate next stages. Moreover, because RT may induce delayed cognitive deteriorations, the onco-functional balance must be extensively discussed by tailoring a real patient-centered attitude (12, 56). The ultimate aim should be to use the good treatment(s) at the optimal moment(s) according not only to the tumor genetics but also other prognostic parameters and patient’s expectations over time. Remarkably, recent series showed that applying this concept led to OS over 16 to 17 years while preserving the QoL for over one decade (10, 11).

## Conclusions

Beyond the fundamental opposition between precision medicine relying on molecular EBM and individualized multistep therapeutic approach adapted over years, “best quality of care” starts by giving the choice to the patient and family and by honestly detailing both philosophies. This approach of complexity is time-consuming and poorly suited to productionist practices of our care systems. It is, nevertheless, possible, independent of the socio-cultural level of each patient, and it represents the condition of a true interactive patient-centered medicine, far from a “unique solution” dogma.

The other risk of a single thought is to disempower the physicians who will not continue to actively discuss the best therapeutic option tailored to each patient but only passively apply a “standardized protocol”. This could lead to an impoverishment of knowledge, failing to see the full picture if all alternatives are not critically considered anymore. The ultimate danger would be to end up with strategies exclusively dictated by processing of large databanks with pre-defined reductive parameters or to use artificial intelligence methods disconnected from clinical practice and real life: this may turn doctors into uncritical executing agents.

Therefore, official recommendations should only be a guide, and tumor boards should provide consultative proposals but not become too oppressive (particularly for medico-legal issues); otherwise, a rigid EBM might kill innovation, which is still essential because glioma patients cannot yet be cured.

In summary, although efforts have been made to excavate different molecular subtypes from the formerly not well-defined mix of gliomas LGG (57, 58), more refined instruments measuring QoL are still lacking. Overcoming the problem of an overbalance of molecular marker can only be counteracted by triggering high-quality multicentric studies focusing on imaging and QoL issues.

## Author Contributions

LT and HD contributed to conception and design of the study. HD wrote the first draft of the manuscript. LT and TO wrote sections of the manuscript. All authors contributed to the article and approved the submitted version.

## Conflict of Interest

The authors declare that the research was conducted in the absence of any commercial or financial relationships that could be construed as a potential conflict of interest.
